# 5,5-Dihydroxy­barbituric acid 1,4-dioxane hemisolvate

**DOI:** 10.1107/S1600536810015321

**Published:** 2010-04-30

**Authors:** Thomas Gelbrich, Denise Rossi, Ulrich J. Griesser

**Affiliations:** a Institute of Pharmacy, University of Innsbruck, Innrain 52, 6020 Innsbruck, Austria

## Abstract

The asymmetric unit of the title compound,, C_4_H_4_N_2_O_5_·0.5C_4_H_8_O_2_, contains one molecule of 5,5-dihydroxybarbituric acid with a nearly planar barbiturate ring and half a molecule of 1,4-dioxane. The geometry of the centrosymmetric dioxane molecule is close to an ideal chair conformation. The crystal structure exhibits a complex three-dimensional hydrogen-bonded network. Barbiturate mol­ecules are connected to one another *via* N—H⋯O=C, O—H⋯O=C and N—H⋯O(hydr­oxy) inter­actions, while the barbituric acid mol­ecule is linked to dioxane by an O—H⋯O contact.

## Related literature

For the crystal structure of unsolvated 5,5-dihydroxy­barbituric acid, see: Singh (1965 ▶); Harrowfield *et al.* (1989 ▶). For the related monohydrate, see Lewis & Tocher (2004*a* ▶). For the related trihydrate, see Mootz & Jeffrey (1965 ▶); Lewis & Tocher (2004*b* ▶). For hydrogen-bond motifs, see: Bernstein *et al.* (1995 ▶).
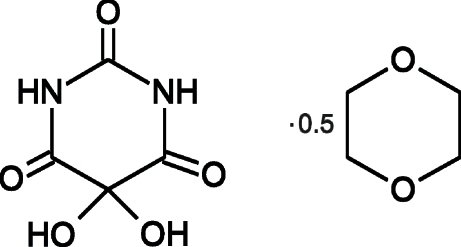


## Experimental

### 

#### Crystal data

C_4_H_4_N_2_O_5_·0.5C_4_H_8_O_2_
*M* *_r_* = 204.14
Triclinic, 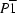

*a* = 6.0232 (3) Å
*b* = 8.3954 (4) Å
*c* = 8.6858 (5) Å
α = 106.007 (4)°
β = 94.459 (3)°
γ = 110.126 (3)°
*V* = 389.09 (3) Å^3^
*Z* = 2
Mo *K*α radiation
μ = 0.16 mm^−1^
*T* = 120 K
0.10 × 0.10 × 0.10 mm

#### Data collection

Bruker-Nonius Roper CCD camera on κ-goniostat diffractometer
Absorption correction: multi-scan (*SADABS*; Sheldrick, 2007 ▶) *T*_min_ = 0.984, *T*_max_ = 0.984
5726 measured reflections
1529 independent reflections
1198 reflections with *I* > 2σ(*I*)
*R*_int_ = 0.049

#### Refinement

*R*[*F* ^2^ > 2σ(*F* ^2^)] = 0.045
*wR*(*F* ^2^) = 0.131
*S* = 1.01
1529 reflections
148 parameters
4 restraints
H atoms treated by a mixture of independent and constrained refinement
Δρ_max_ = 0.25 e Å^−3^
Δρ_min_ = −0.29 e Å^−3^

### 

Data collection: *COLLECT* (Hooft, 1998 ▶); cell refinement: *DENZO* (Otwinowski & Minor, 1997 ▶) and *COLLECT*; data reduction: *DENZO* and *COLLECT*; program(s) used to solve structure: *SHELXS97* (Sheldrick, 2008 ▶); program(s) used to refine structure: *SHELXL97* (Sheldrick, 2008 ▶); molecular graphics: *XP* in *SHELXTL* (Sheldrick, 2008 ▶) and *Mercury* (Macrae *et al.*, 2008 ▶); software used to prepare material for publication: *publCIF* (Westrip, 2010 ▶).

## Supplementary Material

Crystal structure: contains datablocks I, global. DOI: 10.1107/S1600536810015321/jh2150sup1.cif

Structure factors: contains datablocks I. DOI: 10.1107/S1600536810015321/jh2150Isup2.hkl

Additional supplementary materials:  crystallographic information; 3D view; checkCIF report

## Figures and Tables

**Table 1:** Hydrogen-bond geometry (Å, °)

*D*—H⋯*A*	*D*—H	H⋯*A*	*D*⋯*A*	*D*—H⋯*A*
N1—H1*N*⋯O6^i^	0.89 (2)	2.39 (3)	3.068 (2)	134 (3)
N1—H1*N*⋯O7^ii^	0.89 (2)	2.44 (2)	3.180 (2)	141 (3)
N3—H3*N*⋯O2^iii^	0.88 (2)	1.93 (2)	2.810 (2)	172 (2)
O7—H7*O*⋯O1*S*	0.87 (2)	1.87 (2)	2.732 (2)	171 (3)
O8—H8*O*⋯O4^iv^	0.83 (2)	1.95 (2)	2.751 (2)	162 (3)

Symmetry codes: (i) 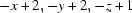; (ii) 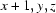; (iii) 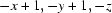; (iv) 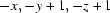.

**Fig. 1.**
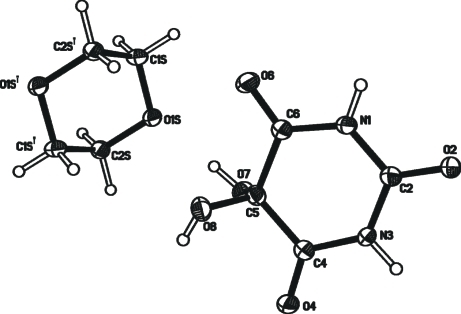
The molecular structures of (I) with displacement ellipsoids drawn at the 50% probability level. Hydrogen atoms are shown as spheres of arbitrary size. Symmetry code: (i) -x+1, -y+2, -z+2.

**Fig. 2.**
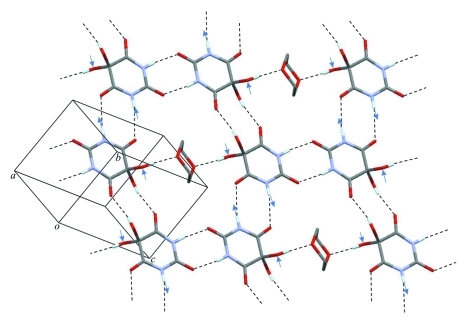
Portion of a hydrogen bonded sheet parallel to (1-21) showing N—H···O=C and O—H···O=C bonds between barbituric acid molecules and O—H···O bonds between barbituric acid and dioxane. N—H···O(hydroxy) interactions linking to two adjacent sheets are indicated by arrows. Dioxane H atoms are omitted for clarity.

## supplementary crystallographic information

### Comment

The crystal structures of an unsolvated form (Singh, 1965; Harrowfield *et al.*, 1989), a monohydrate (Lewis & Tocher, 2004a) and a trihydrate (Mootz & Jeffrey, 1965; Lewis & Tocher, 2004b) of 5,5-dihydroxybarbituric acid have been reported previously. The asymmetric unit of the title structure consists of one molecule of the barbituric acid derivative and one half of a dioxane moiety (Fig. 1). The six-membered C_4_N_2_ ring of the former is essentially planar, and its bond distances and angles are in agreement with the parameters observed for the unsolvated and hydrate forms.

This crystal structure is characterized by extensive hydrogen bonding. Each dihydroxybarbituric acid molecule is linked to two molecules of the same kind via two centrosymmetric N—H···O=C double bridges and a double bridge O—H···O=C connects it to a third molecule. Joining these R^2^_2_(8) and R^2^_2_(10) motifs (Bernstein *et al.*, 1995) gives a larger ring of six dihydroxybarbituric acid molecules. Two molecules of each such ring are additionally O—H···O bonded to a dioxane molecule which lies in the centre of the ring. Fig. 2 shows the 2-dimensional H-bonded net parallel to (121) which is obtained from these interactions. Additionally, one NH and one OH group of each dihydroxybarbituric acid molecule are engaged as H-bond donor and acceptor, respectively, in N—H···O(hydroxy) interactions. These particular contacts, indicated by arrows in Fig. 2, connect adjacent H-bonded 2D units of the kind discussed above to one another, and an overall three-dimensional hydrogen bonded network is therefore formed. As expected, the two hydrogen bonds in which the N1—H group is involved exhibit a much less favourable geometry than the single hydrogen bond in which the N3—H group is employed.

### Experimental

A solution of 5,5-dibromobarbituric acid (Sigma-Aldrich) in dioxane was filled into an NMR tube for a crystallisation experiment by slow evaporation of the solvent. After four months, an amber-coloured syrup had formed, indicating decomposition of the original compound. This liquid contained a large colourless crystal that prooved to be composed of the title compound.

### Refinement

All H atoms were identified in a difference map. H atoms bonded to secondary CH_2_ (C—H = 0.99 Å) carbon atoms were positioned geometrically, and hydrogen atoms attached to N and O were refined with restrained distances [N—H = 0.88 (2) Å, O—H = 0.82 (2) Å]. The *U*_iso_ parameters of all hydrogen atoms were refined freely.

### Crystal data

**Table:**

C_4_H_4_N_2_O_5_·0.5C_4_H_8_O_2_	*Z* = 2
*M**_r_* = 204.14	*F*(000) = 212
Triclinic, *P*1	*D*_x_ = 1.742 Mg m^−^^3^
Hall symbol: -P 1	Mo *K*α radiation, λ = 0.71073 Å
*a* = 6.0232 (3) Å	Cell parameters from 3190 reflections
*b* = 8.3954 (4) Å	θ = 2.9–26.0°
*c* = 8.6858 (5) Å	µ = 0.16 mm^−^^1^
α = 106.007 (4)°	*T* = 120 K
β = 94.459 (3)°	Block, colourless
γ = 110.126 (3)°	0.10 × 0.10 × 0.10 mm
*V* = 389.09 (3) Å^3^	

### Data collection

**Table:**

Bruker-Nonius Roper CCD camera on κ-goniostat diffractometer	1529 independent reflections
Radiation source: Bruker–Nonius FR591 rotating anode	1198 reflections with *I* > 2σ(*I*)
graphite	*R*_int_ = 0.049
Detector resolution: 9.091 pixels mm^-1^	θ_max_ = 26.0°, θ_min_ = 3.6°
φ & ω scans	*h* = −7→7
Absorption correction: multi-scan (*SADABS*; Sheldrick, 2007)	*k* = −10→10
*T*_min_ = 0.984, *T*_max_ = 0.984	*l* = −10→10
5726 measured reflections	

### Refinement

**Table:**

Refinement on *F*^2^	Secondary atom site location: difference Fourier map
Least-squares matrix: full	Hydrogen site location: inferred from neighbouring sites
*R*[*F*^2^ > 2σ(*F*^2^)] = 0.045	H atoms treated by a mixture of independent and constrained refinement
*wR*(*F*^2^) = 0.131	*w* = 1/[σ^2^(*F*_o_^2^) + (0.0787*P*)^2^ + 0.0767*P*] where *P* = (*F*_o_^2^ + 2*F*_c_^2^)/3
*S* = 1.01	(Δ/σ)_max_ < 0.001
1529 reflections	Δρ_max_ = 0.25 e Å^−^^3^
148 parameters	Δρ_min_ = −0.29 e Å^−^^3^
4 restraints	Extinction correction: *SHELXL97* (Sheldrick, 2008), Fc^*^=kFc[1+0.001xFc^2^λ^3^/sin(2θ)]^-1/4^
Primary atom site location: structure-invariant direct methods	Extinction coefficient: 0.062 (16)

### Special details

**Table:**

Geometry. All esds (except the esd in the dihedral angle between two l.s. planes) are estimated using the full covariance matrix. The cell esds are taken into account individually in the estimation of esds in distances, angles and torsion angles; correlations between esds in cell parameters are only used when they are defined by crystal symmetry. An approximate (isotropic) treatment of cell esds is used for estimating esds involving l.s. planes.
Refinement. Refinement of *F*^2^ against ALL reflections. The weighted *R*-factor wR and goodness of fit *S* are based on *F*^2^, conventional *R*-factors *R* are based on *F*, with *F* set to zero for negative *F*^2^. The threshold expression of *F*^2^ > σ(*F*^2^) is used only for calculating *R*-factors(gt) etc. and is not relevant to the choice of reflections for refinement. *R*-factors based on *F*^2^ are statistically about twice as large as those based on *F*, and *R*- factors based on ALL data will be even larger.

### Fractional atomic coordinates and isotropic or equivalent isotropic displacement parameters (Å^2^)

**Table:**

	*x*	*y*	*z*	*U*_iso_*/*U*_eq_	
N1	0.7757 (3)	0.7817 (2)	0.3822 (2)	0.0204 (4)	
H1N	0.934 (3)	0.835 (4)	0.398 (4)	0.061 (10)*	
N3	0.4114 (3)	0.5803 (2)	0.2073 (2)	0.0175 (4)	
H3N	0.342 (4)	0.506 (3)	0.107 (2)	0.029 (6)*	
O2	0.7627 (3)	0.65461 (19)	0.11484 (17)	0.0242 (4)	
O4	0.0683 (3)	0.4836 (2)	0.29980 (18)	0.0264 (4)	
O6	0.8029 (3)	0.90452 (19)	0.65175 (17)	0.0265 (4)	
O7	0.3029 (2)	0.83394 (18)	0.53542 (18)	0.0212 (4)	
H7O	0.360 (6)	0.895 (4)	0.638 (2)	0.064 (10)*	
O8	0.3738 (3)	0.60819 (19)	0.60882 (17)	0.0221 (4)	
H8O	0.228 (3)	0.574 (4)	0.615 (4)	0.060 (10)*	
C2	0.6555 (4)	0.6706 (3)	0.2277 (2)	0.0189 (5)	
C4	0.2802 (4)	0.5806 (3)	0.3280 (2)	0.0185 (5)	
C6	0.6801 (3)	0.8039 (3)	0.5200 (2)	0.0186 (5)	
C5	0.4070 (3)	0.7064 (3)	0.5010 (2)	0.0178 (5)	
O1S	0.4460 (2)	1.04067 (18)	0.85656 (16)	0.0201 (4)	
C1S	0.6766 (4)	1.1366 (3)	0.9647 (3)	0.0218 (5)	
H1S1	0.6742	1.2450	1.0457	0.030 (6)*	
H1S2	0.8027	1.1755	0.9014	0.013 (5)*	
C2S	0.2647 (3)	0.9800 (3)	0.9481 (2)	0.0213 (5)	
H2S1	0.1066	0.9113	0.8733	0.031 (6)*	
H2S2	0.2530	1.0848	1.0287	0.036 (7)*	

### Atomic displacement parameters (Å^2^)

**Table:**

	*U*^11^	*U*^22^	*U*^33^	*U*^12^	*U*^13^	*U*^23^
N1	0.0145 (9)	0.0210 (9)	0.0182 (10)	0.0021 (8)	0.0036 (7)	0.0005 (7)
N3	0.0161 (9)	0.0192 (9)	0.0127 (9)	0.0042 (7)	0.0020 (7)	0.0019 (7)
O2	0.0192 (8)	0.0258 (8)	0.0188 (8)	0.0030 (6)	0.0070 (6)	−0.0003 (6)
O4	0.0159 (8)	0.0307 (8)	0.0211 (8)	0.0004 (7)	0.0042 (6)	0.0016 (6)
O6	0.0191 (8)	0.0319 (9)	0.0186 (8)	0.0056 (7)	0.0014 (6)	−0.0007 (6)
O7	0.0190 (8)	0.0229 (8)	0.0200 (8)	0.0092 (6)	0.0031 (6)	0.0031 (6)
O8	0.0214 (8)	0.0279 (8)	0.0202 (8)	0.0096 (7)	0.0070 (6)	0.0116 (6)
C2	0.0179 (10)	0.0165 (10)	0.0187 (11)	0.0036 (8)	0.0040 (9)	0.0039 (8)
C4	0.0170 (10)	0.0187 (10)	0.0189 (11)	0.0056 (8)	0.0042 (8)	0.0061 (8)
C6	0.0166 (10)	0.0198 (10)	0.0180 (11)	0.0060 (8)	0.0036 (8)	0.0053 (8)
C5	0.0162 (10)	0.0199 (10)	0.0182 (11)	0.0069 (8)	0.0054 (8)	0.0068 (8)
O1S	0.0158 (7)	0.0249 (8)	0.0162 (8)	0.0054 (6)	0.0027 (6)	0.0044 (6)
C1S	0.0152 (10)	0.0225 (10)	0.0205 (11)	0.0019 (8)	−0.0004 (8)	0.0039 (8)
C2S	0.0138 (10)	0.0290 (11)	0.0182 (11)	0.0059 (9)	0.0040 (8)	0.0060 (9)

### Geometric parameters (Å, °)

**Table:**

N1—C6	1.362 (3)	O8—H8O	0.834 (18)
N1—C2	1.374 (3)	C4—C5	1.532 (3)
N1—H1N	0.886 (18)	C6—C5	1.536 (3)
N3—C4	1.361 (3)	O1S—C2S	1.435 (2)
N3—C2	1.373 (3)	O1S—C1S	1.439 (2)
N3—H3N	0.883 (17)	C1S—C2S^i^	1.504 (3)
O2—C2	1.217 (2)	C1S—H1S1	0.9900
O4—C4	1.216 (2)	C1S—H1S2	0.9900
O6—C6	1.214 (2)	C2S—C1S^i^	1.504 (3)
O7—C5	1.394 (2)	C2S—H2S1	0.9900
O7—H7O	0.867 (18)	C2S—H2S2	0.9900
O8—C5	1.392 (2)		
			
C6—N1—C2	126.66 (17)	O8—C5—C4	109.42 (16)
C6—N1—H1N	115 (2)	O7—C5—C4	105.53 (15)
C2—N1—H1N	118 (2)	O8—C5—C6	106.79 (16)
C4—N3—C2	125.94 (17)	O7—C5—C6	108.58 (15)
C4—N3—H3N	119.4 (16)	C4—C5—C6	114.24 (16)
C2—N3—H3N	114.2 (16)	C2S—O1S—C1S	109.37 (15)
C5—O7—H7O	105 (2)	O1S—C1S—C2S^i^	110.63 (16)
C5—O8—H8O	107 (2)	O1S—C1S—H1S1	109.5
O2—C2—N3	122.08 (19)	C2S^i^—C1S—H1S1	109.5
O2—C2—N1	120.85 (18)	O1S—C1S—H1S2	109.5
N3—C2—N1	117.07 (17)	C2S^i^—C1S—H1S2	109.5
O4—C4—N3	121.06 (19)	H1S1—C1S—H1S2	108.1
O4—C4—C5	120.76 (18)	O1S—C2S—C1S^i^	110.95 (16)
N3—C4—C5	118.18 (17)	O1S—C2S—H2S1	109.4
O6—C6—N1	121.57 (18)	C1S^i^—C2S—H2S1	109.4
O6—C6—C5	120.87 (17)	O1S—C2S—H2S2	109.4
N1—C6—C5	117.43 (17)	C1S^i^—C2S—H2S2	109.4
O8—C5—O7	112.40 (16)	H2S1—C2S—H2S2	108.0
			
C4—N3—C2—O2	175.20 (18)	N3—C4—C5—O7	112.76 (19)
C4—N3—C2—N1	−5.1 (3)	O4—C4—C5—C6	173.36 (17)
C6—N1—C2—O2	−176.10 (18)	N3—C4—C5—C6	−6.4 (2)
C6—N1—C2—N3	4.2 (3)	O6—C6—C5—O8	−57.3 (2)
C2—N3—C4—O4	−173.22 (18)	N1—C6—C5—O8	126.73 (18)
C2—N3—C4—C5	6.6 (3)	O6—C6—C5—O7	64.2 (2)
C2—N1—C6—O6	179.18 (18)	N1—C6—C5—O7	−111.85 (19)
C2—N1—C6—C5	−4.8 (3)	O6—C6—C5—C4	−178.39 (17)
O4—C4—C5—O8	53.7 (2)	N1—C6—C5—C4	5.6 (2)
N3—C4—C5—O8	−126.09 (18)	C2S—O1S—C1S—C2S^i^	57.5 (2)
O4—C4—C5—O7	−67.4 (2)	C1S—O1S—C2S—C1S^i^	−57.7 (2)

Symmetry codes: (i) −*x*+1, −*y*+2, −*z*+2.

### Hydrogen-bond geometry (Å, °)

**Table:**

*D*—H···*A*	*D*—H	H···*A*	*D*···*A*	*D*—H···*A*
N1—H1N···O6^ii^	0.89 (2)	2.39 (3)	3.068 (2)	134 (3)
N1—H1N···O7^iii^	0.89 (2)	2.44 (2)	3.180 (2)	141 (3)
N3—H3N···O2^iv^	0.88 (2)	1.93 (2)	2.810 (2)	172 (2)
O7—H7O···O1S	0.87 (2)	1.87 (2)	2.732 (2)	171 (3)
O8—H8O···O4^v^	0.83 (2)	1.95 (2)	2.751 (2)	162 (3)

Symmetry codes: (ii) −*x*+2, −*y*+2, −*z*+1; (iii) *x*+1, *y*, *z*; (iv) −*x*+1, −*y*+1, −*z*; (v) −*x*, −*y*+1, −*z*+1.
